# Supplementary figures and images for: BRITTLE CULM17, a Novel Allele of TAC4, Affects the Mechanical Properties of Rice Plants
Source: Int J Mol Sci. 2022 May 10;23(10):5305. doi: 10.3390/ijms23105305 (PMC9140386; doi:10.3390/ijms23105305)

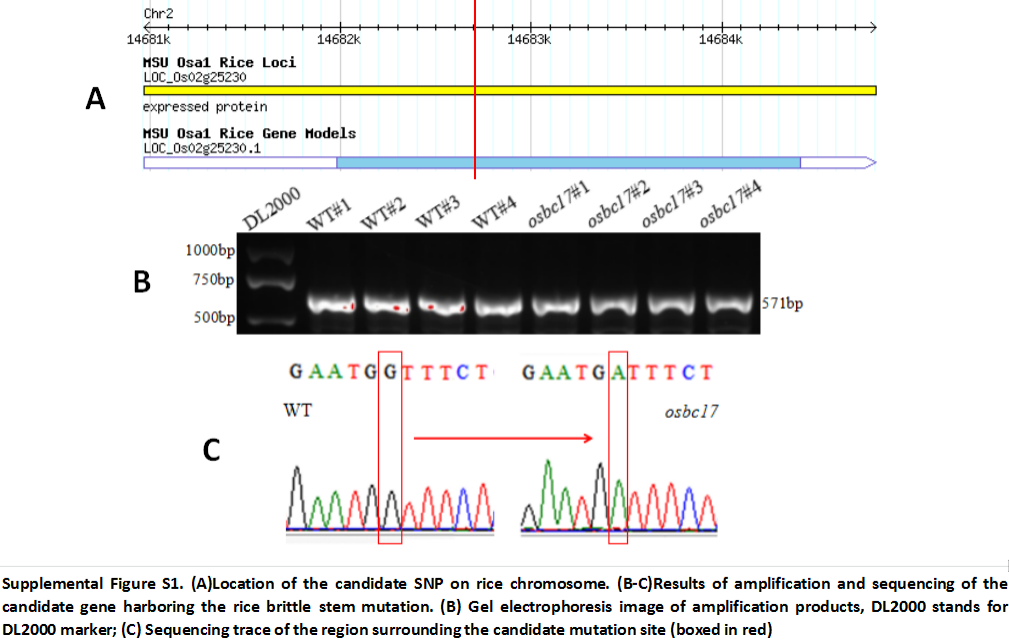

Supplement: Supplementary file 1 [file ijms-23-05305-s001.zip › SupplementalFigureS1.png]

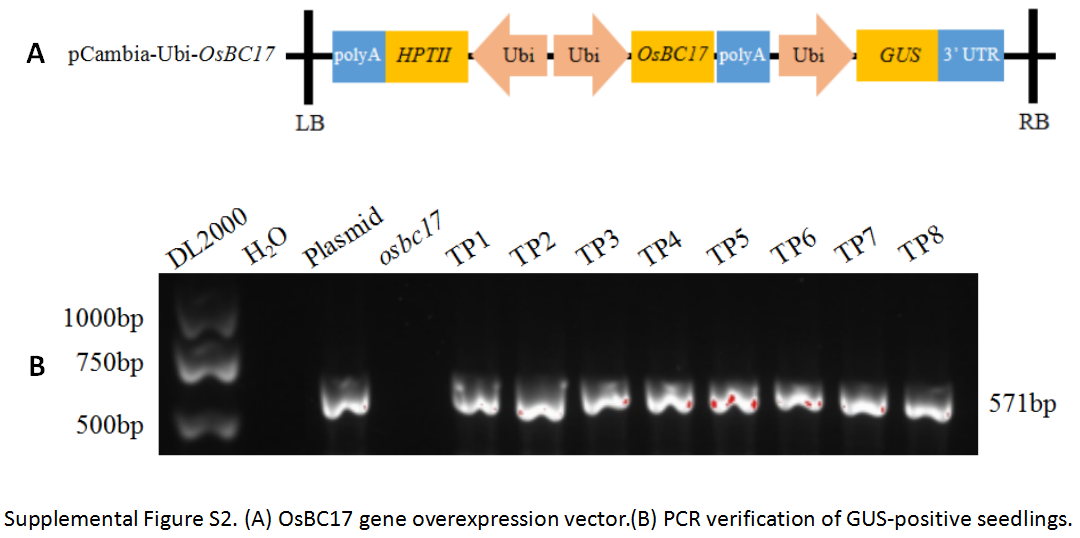

Supplement: Supplementary file 1 [file ijms-23-05305-s001.zip › SupplementalFigureS2.png]

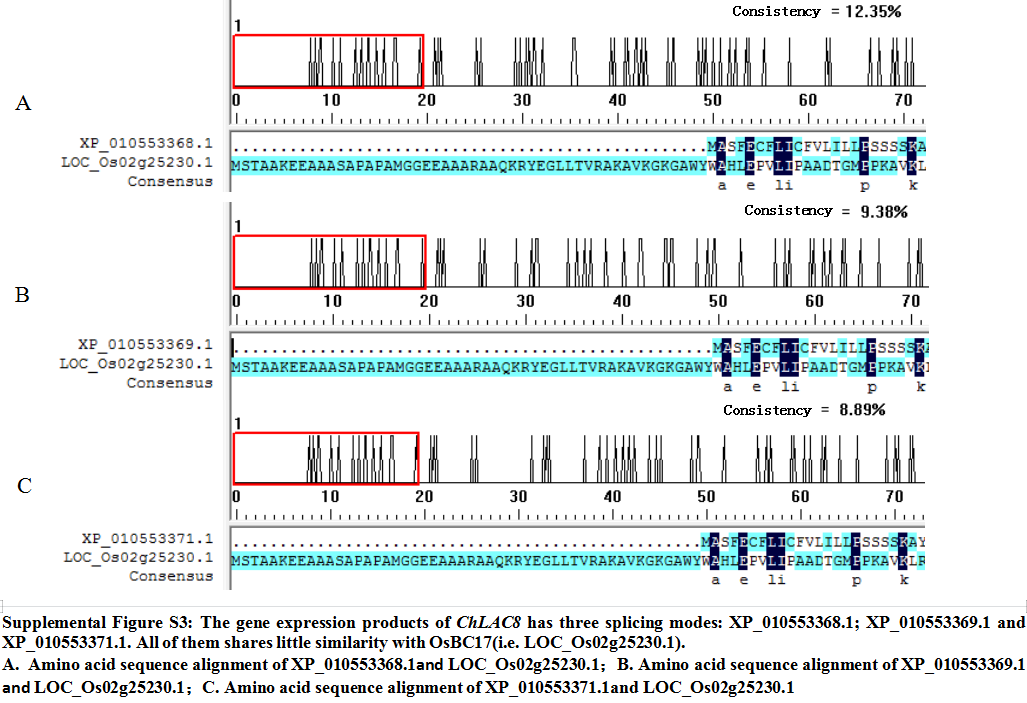

Supplement: Supplementary file 1 [file ijms-23-05305-s001.zip › SupplementalFigureS3.png]

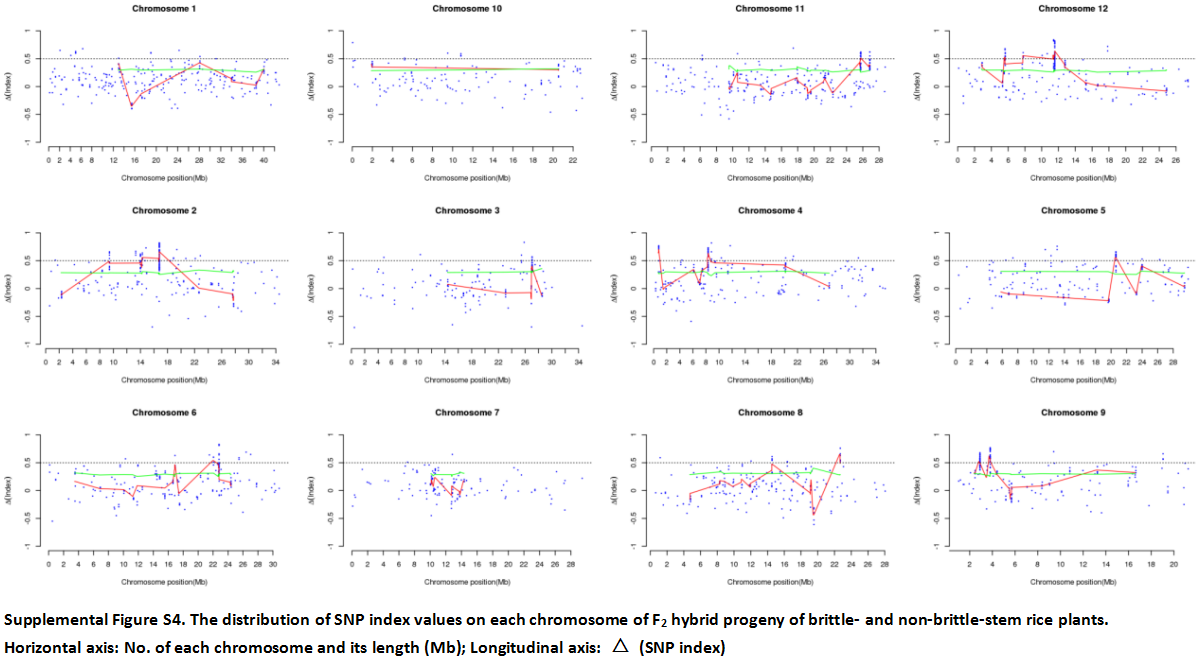

Supplement: Supplementary file 1 [file ijms-23-05305-s001.zip › SupplementalFigureS4.png]
